# Learnable axonal delay in spiking neural networks improves spoken word recognition

**DOI:** 10.3389/fnins.2023.1275944

**Published:** 2023-11-09

**Authors:** Pengfei Sun, Yansong Chua, Paul Devos, Dick Botteldooren

**Affiliations:** ^1^Department of Information Technology, WAVES Research Group, Ghent University, Ghent, Belgium; ^2^Neuromorphic Computing Laboratory, China Nanhu Academy of Electronics and Information Technology, Jiaxing, China

**Keywords:** axonal delay, spiking neural network, speech processing, supervised learning, auditory modeling, neuromorphic computing

## Abstract

Spiking neural networks (SNNs), which are composed of biologically plausible spiking neurons, and combined with bio-physically realistic auditory periphery models, offer a means to explore and understand human auditory processing-especially in tasks where precise timing is essential. However, because of the inherent temporal complexity in spike sequences, the performance of SNNs has remained less competitive compared to artificial neural networks (ANNs). To tackle this challenge, a fundamental research topic is the configuration of spike-timing and the exploration of more intricate architectures. In this work, we demonstrate a learnable axonal delay combined with local skip-connections yields state-of-the-art performance on challenging benchmarks for spoken word recognition. Additionally, we introduce an auxiliary loss term to further enhance accuracy and stability. Experiments on the neuromorphic speech benchmark datasets, NTIDIDIGITS and SHD, show improvements in performance when incorporating our delay module in comparison to vanilla feedforward SNNs. Specifically, with the integration of our delay module, the performance on NTIDIDIGITS and SHD improves by 14% and 18%, respectively. When paired with local skip-connections and the auxiliary loss, our approach surpasses both recurrent and convolutional neural networks, yet uses 10 × fewer parameters for NTIDIDIGITS and 7 × fewer for SHD.

## 1. Introduction

Artificial neural networks (ANNs) have excelled in speech-processing tasks, relying on optimization algorithms, deep architectures, and powerful feature extraction methods like MFCC. Nevertheless, these typical feature extraction methods do not fully replicate the biologically realistic model of cochlear processing (Wu et al., 2018a,b). Additionally, both ANNs and rate-based Spiking Neural Networks (SNNs) struggle with spiking inputs from biologically inspired cochlear models due to their sparse distribution and high temporal complexity (Wu et al., 2021). The high energy consumption of ANNs further limits their deployment in mobile and wearable devices, hindering the development of sound classification systems (Davies et al., 2018; Wu et al., 2018b). Thus, there is a growing demand for bio-inspired SNN architectures capable of handling the outputs of bio-physically realistic cochlear models.

Despite considerable progress in translating insights from non-spiking ANNs to SNNs (Wu et al., 2021; Xu et al., 2023a,b) and the emergence of enhanced architectures (Xu et al., 2018, 2021, 2022) along with sparse training methods (Shen et al., 2023), the primary application has applied to static datasets or non-stream datasets. While earlier research (Mostafa, 2017; Hong et al., 2019; Zhang et al., 2021) has shown encouraging results on such datasets using temporal encoding algorithms, their potential for large-scale time-series datasets remains a question. Contrastingly, noteworthy advancements has been made by algorithms that directly handle event-driven audio tasks with a temporal dimension (Wu et al., 2019, 2020; Zhang et al., 2019; Blouw and Eliasmith, 2020; Yılmaz et al., 2020). A notable method is the refinement of spike timing precision in models and the exploration of intricate architectures that meld both ANN insights and biological understanding. SNNs, which incorporate adjustable membrane and synaptic time constants (Fang et al., 2021; Perez-Nieves et al., 2021), as well as advanced and optimized firing thresholds (Yin et al., 2021; Yu et al., 2022), have shown substantial promise, especially in integrating precise spike timing to achieve top-tier classification accuracy. Although past methods have placed significant emphasis on the importance of spike-timing, believing that information is intricately embedded within the spatio-temporal structure of spike patterns (Wu et al., 2018c), there has been a conspicuous gap in research concerning the specific effects of event transmission, notably axonal delay (Taherkhani et al., 2015). Neurophysiological studies (Carr and Konishi, 1988; Stoelzel et al., 2017) highlight axonal delay's potential role in triggering varied neuronal responses. It is worth noting that axonal delay is a learnable parameter within the brain, extending beyond the realm of synaptic weights (Seidl, 2014; Talidou et al., 2022). Neuromorphic chips such as SpiNNaker (Furber et al., 2014), IBM TrueNorth (Akopyan et al., 2015), and Intel Loihi (Davies et al., 2018) facilitate the programming of the delay module.

These developments have spurred the exploration of jointly training synaptic weights and axonal delay in deep SNNs. While earlier research mainly centered on fixed delays with trainable weights (Bohte et al., 2002) and the concurrent training of synaptic weights and delays in shallow SNNs featuring a single layer (Taherkhani et al., 2015; Wang et al., 2019; Zhang et al., 2020), there has recently been a degree of investigation into the joint training of the synaptic weights and axonal delays in deep SNNs (Shrestha and Orchard, 2018; Shrestha et al., 2022; Sun et al., 2022, 2023a; Hammouamri et al., 2023; Patiño-Saucedo et al., 2023). Our prior effort (Sun et al., 2022) stands as one of the initial successful attempts in applying this method to deep SNNs, achieving promising results in tasks characterized by high temporal complexity.

In this current work, we focus on spiking spoken word recognition tasks, namely NTIDIDIGITS (Anumula et al., 2018) and SHD (Cramer et al., 2020). These tasks are temporally complex (Iyer et al., 2021) and are encoded as spikes through an audio-to-spiking conversion procedure inspired by neurophysiology. In pursuit of enhancing these tasks, we introduce a learnable axonal delay mechanism to govern the transmission process and achieve precise synchronization of spike timing. Alongside the axonal delay module, we delved into various intricate structures, showcasing their synergy with the delay module. Specifically, we propose a novel local skip-connection mechanism designed to mitigate information loss during the reset process, an endeavor that relies heavily on the precise availability of spike timing information. Additionally, we integrate an auxiliary loss to curb unwarranted neuron membrane potentials upon firing. Our results underscore the seamless integration of these intricate components with the delay modules, resulting in substantial performance enhancements. Our methods achieve state-of-the-art performance while requiring fewer parameters, as demonstrated by our experimental studies.

The rest of the paper is organized as follows. We provide a detailed description of the proposed methods in Section 2. In Section 3, we demonstrate the effectiveness of our algorithms on two event-based audio data-sets and compare them with other SNNs and ANNs. We conclude and discuss future work in Section 4.

## 2. Materials and methods

In this section, we begin by introducing the spiking neuron model utilized in this work. After that, we present the Variable Axonal Delay (VAD) and Local Skip-Connection methods. The introduction of the Variable Axonal Delay is loosely inspired by neurophysiology, as we argue that the variation of delays observed in the biological system could be advantageous for aligning temporal information on a millisecond time scale. As a result, transient sensory inputs can be condensed into specific spike bursts corresponding to their transience. Next, we introduce the concept of a local skip-connection architecture, which holds the potential to mitigate information loss during the reset mechanism, thereby enhancing the dynamic behavior of the neuron model. Finally, we demonstrate that the suppressed loss further enhances performance, improving the network's discriminative capabilities for target differentiation.

### 2.1. Spiking neuron model

An SNN employs a spiking neuron as the basic computational unit with input and output in the form of spikes, maintaining an internal membrane potential over time. In this paper, we adopt the Spike Response Model (SRM) which phenomenologically describes the dynamic response of biological neurons.

Consider an input spike, $s_{j}^{l-1}\left(t\right)=\delta \left(t-t_{j}^{\left(l-1\right)}\right)$. Here $t_{j}^{\left(l-1\right)}$ denotes a firing time of pre-synaptic neuron *j* in layer *l* − 1 and δ the spike function. In the SRM model, the incoming spike $s_{j}^{l-1}\left(t\right)$ is converted into spike response signals by convolving with the spike response kernel ϵ(*t*) and is then scaled by the synaptic weight to generate the Post Synaptic Potential (PSP). Likewise, the refractory period can be represented as $\left(\nu *s_{j}^{l}\right)\left(t\right)$ which describes the characteristic recovery time needed before the neuron regains its capacity to fire again after having fired at time *t*. The neuron's membrane potential, is the sum of all PSPs and refractory response

(1) $$\begin{array}{l} u_{i}^{l}\left(t\right)=\underset{j}{\sum }W_{ij}^{l-1}\left(\epsilon *s_{j}^{l-1}\right)\left(t\right)+\left(\nu *s_{i}^{l}\right)\left(t\right) \end{array}$$

where $u_{i}^{l}\left(t\right)$ is the membrane potential of neuron *i* and $W_{ij}^{l-1}$ is the synaptic weight from neuron *j* to neuron *i*.

A firing output is generated wherever *u*_*i*_(*t*) crosses the predefined firing threshold θ_*u*_. This generation process can be formulated by a Heaviside step function Θ as follows

(2) $$\begin{array}{l} s_{i}^{l}\left(t\right)=\Theta \left(u_{i}^{l}\left(t\right)-\theta_{u}\right). \end{array}$$

### 2.2. Variable axonal delay (VAD) module

**Figure 1:**
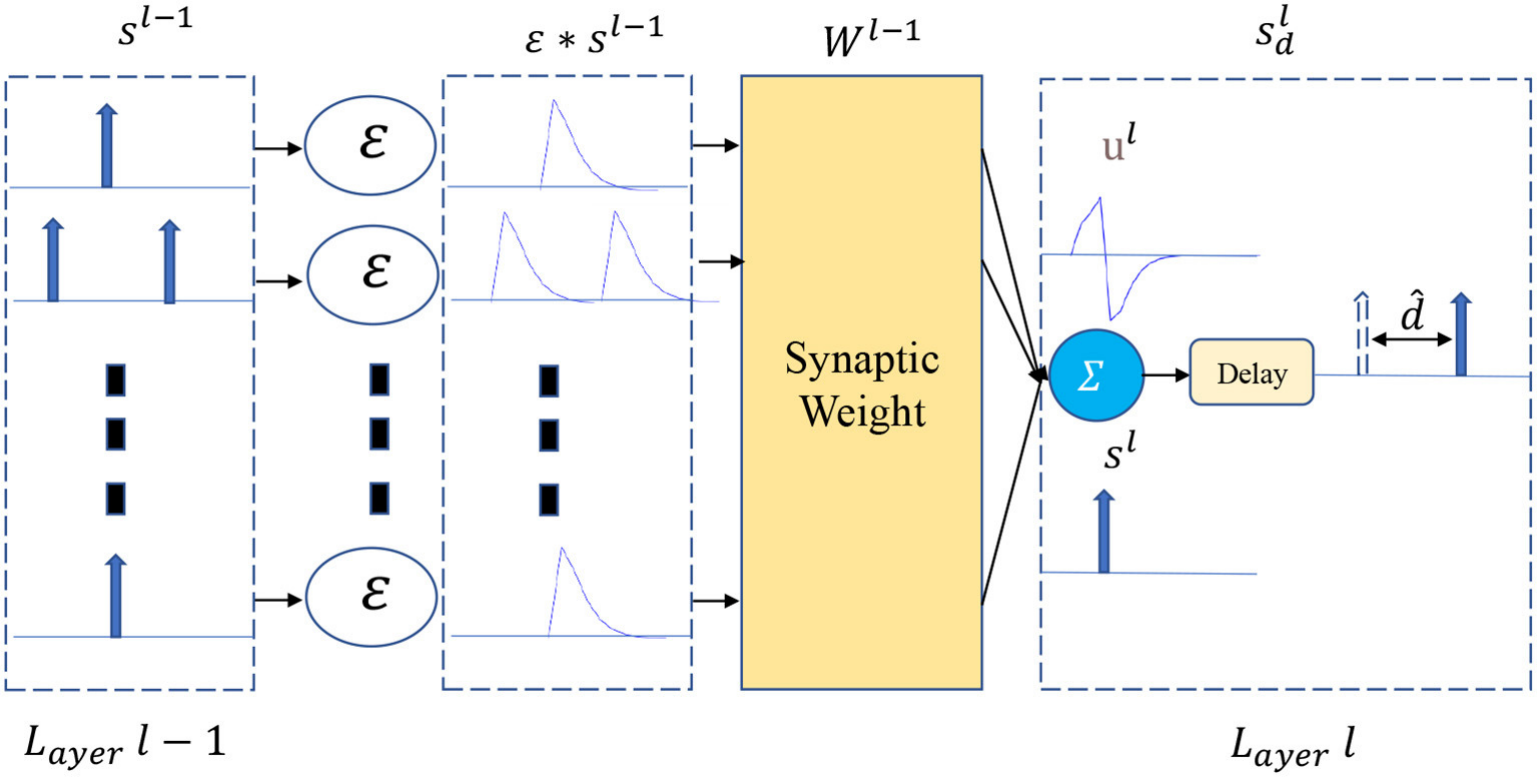
Illustration of the flow chart of the axonal delay. The output spike is delayed by $\hat{d}$.

As shown in Figure 1, a VAD is added to the output of each spiking neuron in layer *l*. Let *N* be the number of neurons at layer *l*, thus, the set of spike trains *s*^*l*^(*t*) can be represented as follows

(3) $$\begin{array}{l} s^{l}\left(t\right)=\left\{s_{1}^{l}\left(t\right),...,s_{N}^{l}\left(t\right)\right\} \end{array}$$

The forward pass of the delay module can be described as

(4) $$\begin{array}{l} s_{d}^{l}\left(t\right)=\delta \left(t-\hat{d^{l}}\right)*s^{l}\left(t\right) \end{array}$$

Where ${\hat{d}}^{l}$ is the set of learnable delays $\left\{{\hat{d}}_{1},{\hat{d}}_{2},..,{\hat{d}}_{N}\right\}$ in layer *l*, and $s_{d}^{l}\left(t\right)$ is the spike trains output by the delay module. From the system point of view, limiting the axonal delay of each neuron to a reasonable range can speed up the training convergence. Thus, we clip the delay to the specified range during training and round down after each backpropagation.

(5) $$\begin{array}{l} {\hat{d}}^{l}=Min\left(Max\left(0,round\left(\hat{d}\right)\right),\theta_{d}\right) \end{array}$$

Here, the θ_*d*_ refers to the upper bound of the time delay of the spiking neuron.

### 2.3. Local skip-connection as compensation for loss of information due to reset

**Figure 2:**
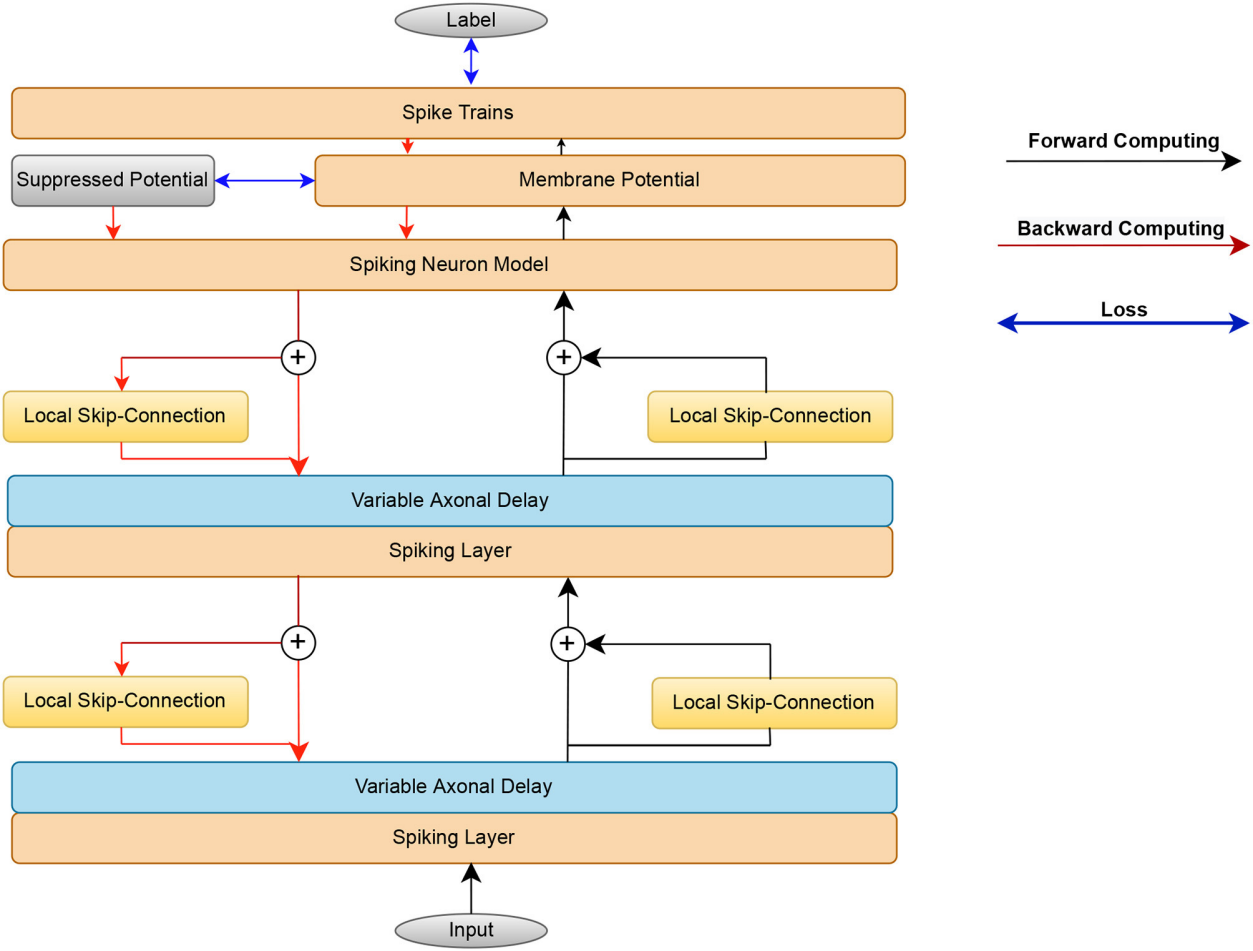
Flow chart illustrating the proposed methods. In the forward pass, the input spikes are mapped by the SRM, axonal delay module, and local skip-connection to the output spikes. The error consists of the spike rate loss from the last layer and the suppressed loss from the false neuron's membrane potential. The spiking layer consists of the Spiking neuron model and membrane potential layer. The error gradients are passed backward through time to update the weight and axonal delay parameters.

The structure of the local skip-connection within a given layer is depicted in Figure 2. In mapping from input spikes to output spikes, The SRM utilizes a refractory kernel to characterize the refractory mechanism, represented by the equation $\nu \left(t\right)=-\alpha_{r}\theta_{u}\frac{t}{\tau_{r}}\exp\left(1-\frac{t}{\tau_{r}}\right)\Theta \left(t\right)$. One challenge that persists is identifying the ideal refractory scale α_*r*_ for specific tasks. If the refractory scale is too small, its effect is diminished, while an overly large refractory scale risks information loss at certain time junctures. To address this, our study introduces the concept of a local skip-connection. This design compensates for information lost during the reset mechanism in a dynamic fashion. Our results show that this connection can operate effectively using the same refractory scale, offering a solution to the intricate task of selecting an optimal refractory scale for various tasks. The output membrane potential of the local skip-connection can be formulated as

(6) $$\begin{array}{l} {û}_{i}^{l}\left(t\right)=\underset{j}{\sum }V_{ij}^{l}\left(\epsilon *s_{d,j}^{l}\right)\left(t\right)+\left(\nu *{ŝ}_{i}^{l}\right)\left(t\right) \end{array}$$

$V_{ij}^{l}$ is the locally connected synaptic weight from neuron *j* to neuron *i* at the same layer. Unlike a skip connection, the local skip-connection adds an extra layer of processing to the output spikes generated in layer *l*. It then directs these locally processed output spikes, denoted as ŝ^*l*^ with the same index as the original output spikes $s_{d}^{l}$, to follow the same axon line within layer *l*. As a result, both the local spike trains ŝ^*l*^ and the original output spikes $s_{d}^{l}$ utilize the same weights $W_{ij}^{l}$ and are channeled to the succeeding layer. This can be equivalently expressed as $s^{l}=s_{d}^{l}+{ŝ}^{l}$.

### 2.4. Loss layer

The loss of an SNN compares the output spikes with the ground truth. However, in classification tasks, decisions are typically made based on the spike due to the absence of precise timing. Considering the spike rate over the time interval *T*, the loss function *L* can be formulated as follows:

(7) $$\begin{array}{l} L=\frac{1}{2}{\left(\int_{0}^{T}\tilde{s}\left(\tau \right)d\tau -\int_{0}^{T}s^{n_{l}}\left(\tau \right)d\tau \right)}^{2} \end{array}$$

Here, *L* measures the disparity between the target spike train $\tilde{s}\left(t\right)$ and output spike train *s*^*nl*^(*t*) at the last layer *n*_*l*_ across the simulation time *T*. Given the lack of precise spike timing in our tasks, we measure the output spikes through the integration of $s^{n_{l}}\left(t\right)$ over *T*. For different task scenarios, the target firing rate is set as $\int_{0}^{T}\tilde{s}\left(\tau \right)d\tau$.

To further exploit temporal information in classification, an auxiliary loss termed the suppressed loss *L*_*Mem*_ is introduced:

(8) $$\begin{array}{l} L_{Mem}\left(t\right)=\frac{1}{2}\cdot {\left(s^{n_{l}}\left(t\right)\cdot Mask\cdot \left(u^{n_{l}}\left(t-\Delta t\right)-u_{\theta }\right)\right)}^{2} \end{array}$$

This loss function is designed to reduce the firing probability of incorrect neurons right when they activate. Compared to previous lateral inhibition methods using learnable or fixed kernels, this loss function achieves a winner-takes-all effect by acting as a regularizer. Importantly, this loss is only applied to false neurons. Here, the spike train $s^{n_{l}}$ and membrane potential $u^{n_{l}}$ are functions of time. Moreover, $u^{n_{l}}\left(t-\Delta t\right)$ refers to the membrane potential right before a spike occurs. When a neuron is activated, indicated by $s^{n_{l}}\left(t\right)=1$, its potential is referred to as $u^{n_{l}}\left(t-\Delta t\right)$. This value is then subtracted from a predetermined membrane potential *u*_θ_, controlled by the suppressing factor λ_*u*_ and defined as *u*_θ_ = λ_*u*_θ_*u*_. Lastly, to ensure that the suppressed membrane potential loss is limited only to undesired (or false) neurons, a mask *Mask* ∈ ℝ^*C*^ is employed, where *C* is the number of target neurons:

(9) $$\begin{array}{l} Mask=\{\begin{array}{ll} 0 & \text{True Class}\\ 1 & \text{False Classes} \end{array} \end{array}$$

### 2.5. Backpropagation

The surrogate gradient algorithm in combination with the Backpropagation-Through-Time (BPTT) (Werbos, 1990) in SNN has shown excellent performance on temporal pattern recognition tasks.

In this work, we discretise the temporal dimension with the sampling time *T*_*s*_ such that *t* = *nT*_*s*_ where *n* denotes the time step of the simulation. We also define (*N*_*s*_ + 1)*T*_*s*_ as the total observation time. For the Heaviside step function, we adapt the SLayer function (Shrestha and Orchard, 2018) to formulate the proxy gradient, which is defined as

(10) $$\begin{array}{l} \hat{f_{s}^{{\;}^{\prime }}}=\tau_{scale}\exp\left(-|u\left(t\right)-\vartheta |/\tau_{\vartheta }\right) \end{array}$$

Here, τ_*scale*_ and τ_ϑ_ are two parameters that control the sharpness of the surrogate gradient. Similarly, the gradient of the axonal delay is given by

(11) $$\begin{array}{l} \nabla_{{\hat{d}}^{l}}E=T_{s}\overset{N_{s}}{\underset{n=0}{\sum }}\frac{\partial L\left[n\right]}{\partial {\hat{d}}^{l}} \end{array}$$

Using the chain rule and noting that the loss at time-step *n* depends on all previous timesteps, we get

(12) $$\begin{array}{l} \begin{array}{cc} \nabla_{{\hat{d}}^{l}}E & =T_{s}\overset{N_{s}}{\underset{n=0}{\sum }}\overset{n}{\underset{m=0}{\sum }}\frac{\partial s_{d}^{l}\left[m\right]}{\partial d^{l}}\frac{\partial L\left[n\right]}{\partial s_{d}^{l}\left[m\right]}\\ =T_{s}\overset{N_{s}}{\underset{n=0}{\sum }}\overset{n}{\underset{m=0}{\sum }}\frac{s_{d}^{l}\left[m\right]-s_{d}^{l}\left[m-1\right]}{T_{s}}\frac{\partial L\left[n\right]}{\partial s_{d}^{l}\left[m\right]} \end{array} \end{array}$$

Here, the finite difference approximation $\frac{s_{d}^{l}\left[m\right]-s_{d}^{l}\left[m-1\right]}{T_{s}}$ is used to numerically estimate the gradient term $\frac{\partial s_{d}^{l}\left[m\right]}{\partial d^{l}}$. As part of the backpropagation process, the gradient of delay is propagated backward, and then the delay value is subsequently updated. Similarly, we also formulate the gradient term of the suppressed loss.

(13) $$\begin{array}{ll} \;\frac{\partial L_{Mem}}{\partial u^{n_{l}}} & =s^{n_{l}}\cdot Mask\cdot \left(u^{nl}-u_{\theta }\right) \end{array}$$

As shown in Figure 2, beginning from the input layer, the spike trains compute forward and the error propagates backward.

## 3. Experiments and results

In this section, we first evaluate the effectiveness of the proposed delay module and novel architecture on two event-based audio datasets: NTIDIDIGITS and SHD. Additionally, we assess the impact of the novel auxiliary loss in boosting performance. Finally, we compare our results with several state-of-the-art networks, including feedforward SNNs, recurrently connected SNNs (RSNNs), and Recurrent Neural Networks (RNNs).

### 3.1. Implementation details

The experiments are conducted using PyTorch as a framework, and all reported results are obtained on 1 NVIDIA Titan XP GPU. Each network and proposed architecture is trained with the Adam optimizer (Kingma and Ba, 2014) and has the same training cycle. The simulation time step *T*_*s*_ is 1 ms, and the firing threshold θ_*u*_ is set at 10 mV. The chosen response kernel is $\epsilon \left(t\right)=\frac{t}{\tau_{s}}\exp\left(1-\frac{t}{\tau_{s}}\right)\Theta \left(t\right)$, and the refractory kernel is $\nu \left(t\right)=-\alpha_{r}\theta_{u}\frac{t}{\tau_{r}}\exp\left(1-\frac{t}{\tau_{r}}\right)\Theta \left(t\right)$. The time constant of the response kernel τ_*s*_ and refractory kernel τ_*r*_ is set to 5 for NTIDIDIGITS and 1 for SHD datasets. The suppressed factor λ_*u*_ is set to 0.995 to suppress the membrane potential of the firing undesired neurons below the threshold. For the proxy gradient, we adopt the Slayer (Shrestha and Orchard, 2018). Table 1 lists other hyperparameters used.

**Table 1:** Detailed hyper-parameter settings.

**Hyper-parameter**	**N-TDIDIGITS18**	**SHD**
Batch size	128	128
Learning rate	0.1	0.1
Time constant τ_*s*_	5	1
Time constant τ_*r*_	5	1
Membrane threshold θ_*u*_	10	10
Refractory scale α_*r*_	2	2
Delay threshold θ_*d*_	128	64
Suppressed factor λ_*u*_	0.995	0.995

The following notation is used to describe the network architecture: “FC” stands for a fully-connected layer, “VAD” means Variable Axonal Delay module, “Local” denotes the local skip-connection architecture, and *L*_*Mem*_ implies the use of the suppressed loss in addition to the spike rate loss. For example, Input-128FC-VAD-Local-128FC-VAD-Local-Output + L_{Mem} indicates that there are two dense layers with 128 neurons, each implementing the VAD and Local module. The loss is measured by the spike rate and suppressed membrane potential. Table 2 summarizes the abbreviations for different architectures and methods.

**Table 2:** Name and corresponding network structure. L2 denotes the l2 regularizer for delay values.

**Name**	**Network structure**
D128-SNN	Input-128FC-VAD-128FC-VAD-Output
DL128-SNN	Input-128FC-VAD-Local-128FC- VAD-Local-Output
DL128-SNN-Dloss	Input-128FC-VAD-Local-128FC-VAD- Local-Output + *L*_*Mem*_
DL256-SNN-Dloss	Input-128FC-VAD-Local-256FC-VAD- Local-Output + *L*_*Mem*_
DL128-SNN-Dloss-L2	Input-128FC-L2(VAD)-Local-128FC-L2(VAD)- Local-Output + *L*_*Mem*_

The number of spikes generated from the last layer is compared to the desired spikes in dedicated output nodes, serving as the primary loss measurement. In order to implement the suppressed membrane potential loss function, the model is pre-trained for 20 epochs to generate the target spike trains used for *L*_*Mem*_ definition. For a fair comparison, all the experiments are run for 5 independent trials, and the average performance and standard deviation are reported.

### 3.2. Datasets

Tests are performed on the speech classification datasets NTIDIDIGITS and Spiking Heidelberg Digits (SHD). Both datasets represent events in the form of spikes, containing rich temporal information that is naturally suited to be directly processed by an SNN. These datasets are considered benchmarks, allowing us to focus on the architecture and learning algorithm of the SNN without considering the spike generation method.

#### 3.2.1. NTIDIDIGITS

**Figure 3:**
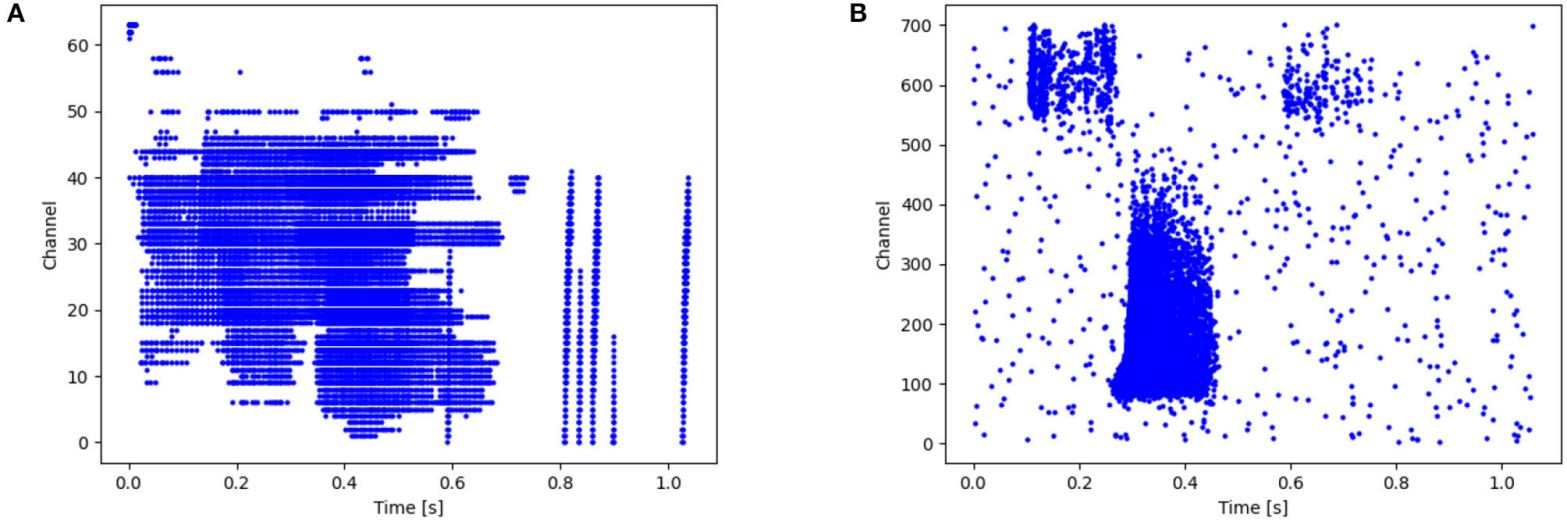
**(A)** Raster plot of Spiketrains of input from a single sample (label 0) of the NTIDIDIGITS dataset. The y-axis represents the channels of the cochlear model while the x-axis indicates the time. **(B)** An Illustration of one raw example (word “six”) from the SHD dataset.

The NTIDIDIGITS (Anumula et al., 2018) dataset was created by playing the TDIDIGITS (Leonard and Doddington, 1993) to the 64 response channel silicon cochlea. The dataset includes single digits and connected digit sequences, all of which contain the 11 spoken digits (“oh,” and the digits “0” to “9”). For the n-way classification problem (single digits), there are a total of 55 male and 56 female speakers with 2,463 training samples, and 56 male and 53 female speakers in the testing set with a total of 2,486 samples. As shown in Figure 3A, the time resolution is in *ms* level and the channel ranges from 0 to 63.

#### 3.2.2. SHD

The SHD is the spiking version of the Heidelberg Digits (HD) audio dataset that is converted by a biologically inspired cochlea model (Cramer et al., 2020). There are 8,156 and 2,264 spoken samples for training and testing, respectively. It contains 10-digit utterances from “0” to “9” in English and German, with a total of 20 classes presented by 12 speakers. Figure 3B shows an example of this audio spike stream. Each sample duration ranges from 0.24 to 1.17 s. Here, the time is resampled to speed up the training (Yin et al., 2020). Each channel has at most 1 spike every 4 *ms* and shorter samples are padded with zeros.

### 3.3. Overall results

This section demonstrates the benefits of the proposed innovations and assesses the effects of the VAD, Local skip-connection, and Suppressed loss individually to validate their impact on boosting performance. The basic SNN consists of 2 hidden layers, followed by the VAD module, Local skip-connection in each layer, and the suppressed loss module in the readout layer's membrane potential (Figure 2).

1) NTIDIDIGITS. As shown in Table 3, non-spiking approaches such as GRU-RNN and Phased-LSTM (Anumula et al., 2018) achieve 90.90 and 91.25% accuracy, respectively. However, these RNNs rely on the event synthesis algorithm and cannot fully exploit sparse event-based information. Zhang and Li (2019) directly train the spike-train level features with recurrent layers through the ST-RSBP method, and Zhang and Li (2021) further propose the SrSc-SNNs architectures that consist of three self-recurrent layers with skip-connections, training this SNN using backpropagation-based intrinsic plasticity, achieving state-of-the-art (SOTA) performance. We show that with the proposed VAD module, local skip-connection, and suppressed loss, our method achieves 95.30% accuracy with a mean of 95.22% and a standard deviation of 0.08%, making it the best result in this classification task. Furthermore, our model uses the least parameters and is 10× smaller compared to the second-best result.

**Table 3:** Comparison of classification and parameter count of proposed methods on the NTIDIDIGITS and SHD Test sets.

**Dataset**	**Method**	**Params**	**Accuracy (%)**
N-TDIDIGITS18	GRU-RNN (Anumula et al., 2018)^†^	0.11M	90.90
	Phased-LSTM (Anumula et al., 2018)^†^	0.61M	91.25
	ST-RSBP (Zhang and Li, 2019)	0.35M	93.90
	SrSc-SNNs-IP (Zhang and Li, 2021)	0.61M	95.07
	**DL128-SNN-Dloss**	**0.06M**	**95.22**
SHD	Feed-forward SNN (Cramer et al., 2020)	0.09M	48.1
	RSNN (Cramer et al., 2020)	1.79M	83.2
	RSNN with adaption (Yin et al., 2020)	0.14M	84.40
	Heterogeneous RSNN (Perez-Nieves et al., 2021)	0.11M	82.78
	RSNN with attention (Yao et al., 2021)	0.14M	91.08
	DMUC (Sun et al., 2023b)^†^	0.24 M	91.48%
	CNN (Cramer et al., 2020)^†^	1.01M	92.40
	RadLIF (Bittar and Garner, 2022)	3.9M	94.62
	DCLS (Hammouamri et al., 2023)^*^	0.21M	**95.07**
	SNN with delays (Patiño-Saucedo et al., 2023)	0.1M	90.04
	**DL128-SNN-Dloss**	0.14M	92.56
	**DL256-SNN-Dloss**	0.21M	93.55

† Non-SNN implementation.

* Channel reduction. Bold values are the best results.

2) SHD. For this dataset, we compare our methods with recent advancements. In Cramer et al. (2020), the single feed-forward SNN and Recurrent SNN are both trained using BPTT. Their results show that the recurrent architecture outperforms the homogeneous feed-forward architecture in this challenging work, underscoring the potential advantages of intricate SNN designs. Several studies have ventured into specialized SNN architectures. For instance, some explore the effectiveness of the heterogeneous recurrent SNNs (Perez-Nieves et al., 2021), while others delved into attention-based SNNs (Yao et al., 2021). As detailed in Table 3, our proposed method produces a competitive performance of 92.56% in a two-layer fully connected network of 128 neurons each. Notably, this performance is competitive compared to these results that employ the same data processing methods and network architecture. Patiño-Saucedo et al. (2023) introduce axonal delays in tandem with learnable time constants, enabling a reduction in model size to a mere 0.1 M while preserving competitive performance.

Additionally, RadLIF (Bittar and Garner, 2022) combines an adaptive linear LIF neuron with the SG strategy, achieving a performance of 94.62%. This achievement is realized through the utilization of three recurrent spiking layers, each containing 1024 neurons. On the other hand, DCLS, introduced in Hammouamri et al.'s research (Hammouamri et al., 2023), capitalizes on several key innovations. It incorporates learnable position adjustments within the kernel, employs advanced data augmentation techniques (like the 5-channel binning), and incorporates batch normalization methods. As a result, DCLS achieves an accuracy of 95.07% using two feedforward spiking layers, each comprising 256 neurons. Given the sizeable 700-input channel, we mitigated extensive parameter expansion by augmenting the neural network's second layer from 128 to 256 neurons. This strategic adjustment significantly improved performance, yielding a 93.55% accuracy rate.

### 3.4. Ablation study

We delve into the contributions of VAD, Local skip-connection, and Suppressed loss via a comprehensive ablation study (refer to Table 4). Evaluating each method individually on two fully-connected feed-forward SNNs provides the following insights:

- **VAD:** When incorporated, there is a marked enhancement in the accuracy across datasets. Specifically, with the delay module embedded (in the D128-SNN setup), we obtain gains of 14.47% and 18.68% for NTIDIDIGITS and SHD, respectively. Importantly, despite these advancements, the parameters remain nearly unchanged. This is attributed to our adoption of channel-wise delays, implying that the increase in parameters corresponds only to the number of channels in each layer. As an illustration, with the SHD dataset, the integration of VAD results in an increment of *N* parameters in each layer, with *N* being set to 128 in our experimental setup.
- **Local skip-connection:** Its standalone application (reflected in the Input-128FC-Local-128FC-Local-11 design) does not bolster accuracy notably. For the SHD dataset, the outcome is even slightly detrimental. However, this method increases the number of trainable parameters. This can be likened to the addition of an extra feedforward layer, resulting in a parameter increment of *N* × *N* for each layer.

**Table 4:** Ablation studies for different architecture and learning methods.

**Dataset**	**Network**	**Params**	**Accuracy (%)**
NTIDIDIGITS	Input-128FC-128FC-11	26,251	78.52
	Input-128FC-Local-128FC-Local-11	59,275	79.36
	D128-SNN	26,507	92.99
	DL128-SNN	59,531	94.70 ± 0.35
	DL128-SNN-Dloss	59,531	95.22 ± 0.08
	DL128-SNN-Dloss-L2	59,531	94.85 ± 0.08
SHD	Input-128FC-128FC-20	108,820	67.05
	Input-128FC-Local-128FC-Local-20	141,844	65.55
	D128-SNN	109,076	85.73
	DL128-SNN	142,100	91.52 ± 0.84
	DL128-SNN-Dloss	142,100	92.56 ± 0.56
	DL128-SNN-Dloss-L2	142,100	92.44 ± 0.09

Combining VAD and Local skip-connection in the DL128-SNN design yields significant benefits. We clinch state-of-the-art accuracy levels for both datasets. This highlights that the enhanced flexibility provided by VAD truly shines when paired with a richer parameter landscape, as provided by the Local skip-connection. Lastly, supplementing the above with the suppressed loss, Dloss, results in stellar performance: 95.22% for NTIDIDIGITS and 92.56% for SHD.

### 3.5. Axonal delay improves the characterization learning ability

**Figure 4:**
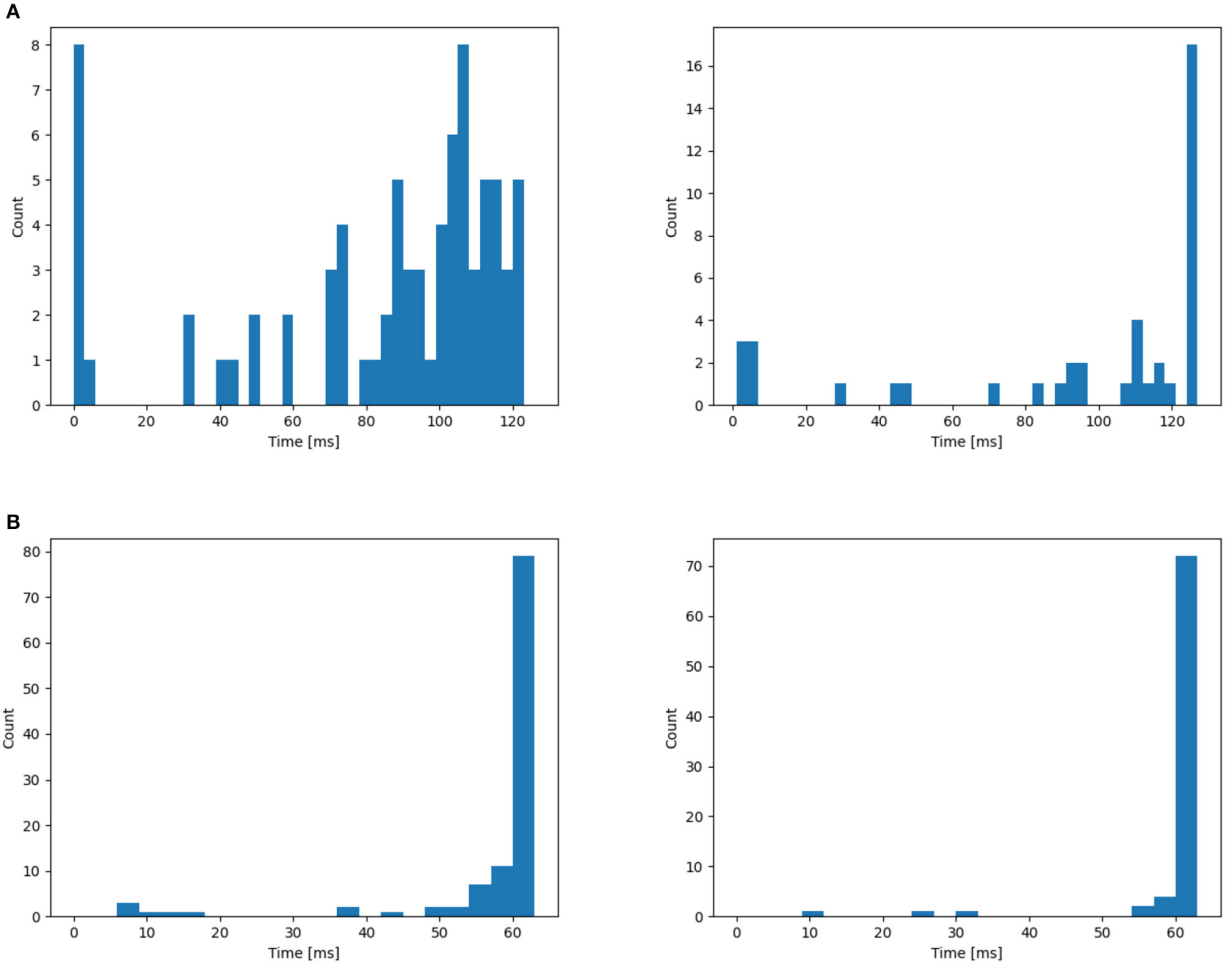
Distribution of time delay on **(A)** NTIDIDIGITS, **(B)** SHD. The initial distribution are all 0. From the left to right: First layer, second layer.

In this section, we begin by offering a visual representation of the axonal delay distribution (refer to Figure 4) for both datasets. Subsequently, we employ an L2 regularizer on the delay to curtail the magnitude of delay values, effectively reducing the number of delayed time steps.

**Figure 5:**
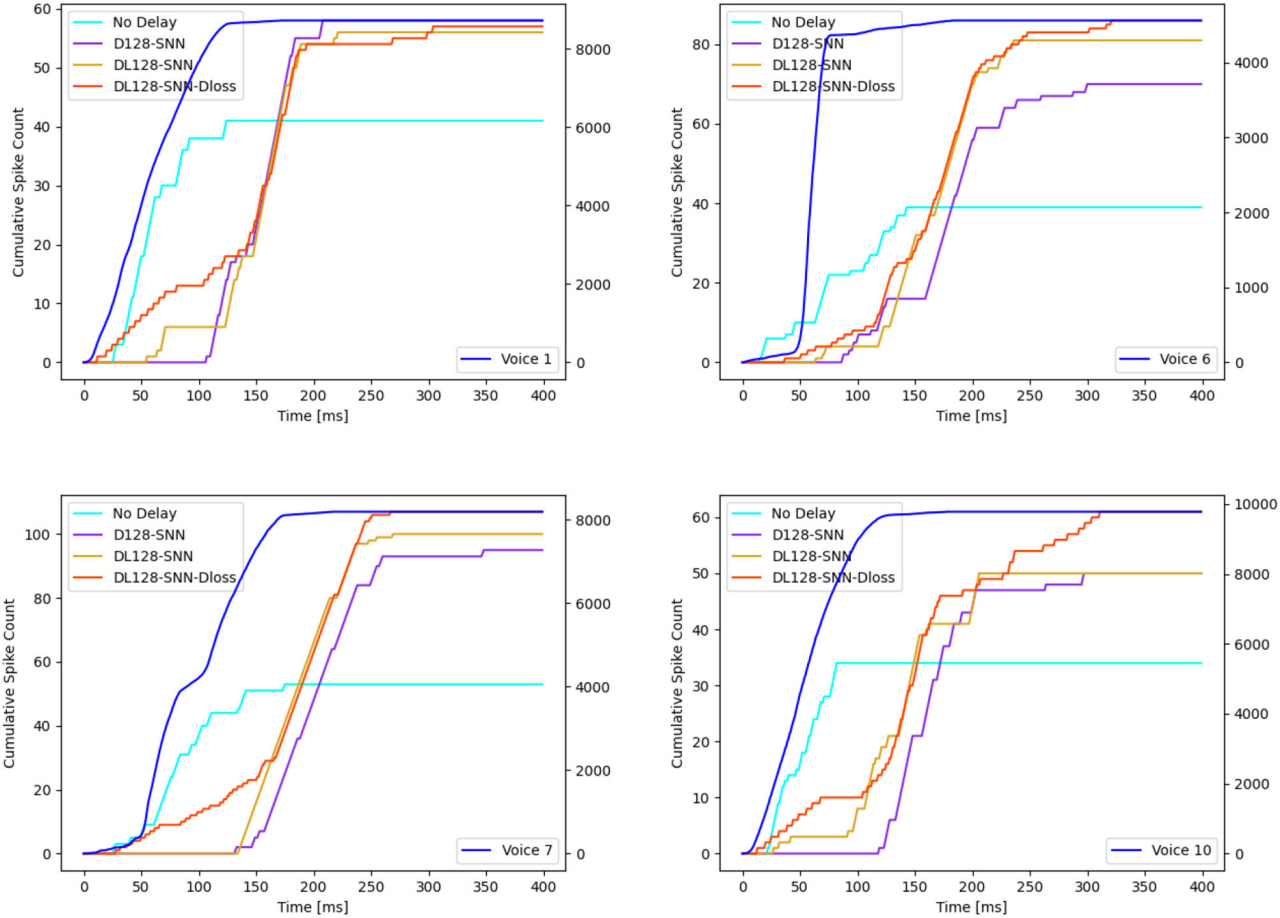
Illustration of 4 distinct English examples (“1”, “6”, “7”, and “10”). The cumulative spike count of the input is plotted on the right y-axis (represented by the blue line), while the true neurons' cumulative spike count is on the left y-axis. Four models are showcased:: No delay, D128-SNN, DL128-SNN, and DL128-SNN-Dloss.

Utilizing the NTIDIDIGITS dataset as an illustrative example, Figure 4A reveals a delay distribution in the first layer that consistently encompasses both long and short delay neurons. This may imply that certain neurons focus on the initial portion of the input, whereas others concentrate on the latter segment of the input features. To understand the dynamics of the VAD, we inspect the cumulative spike count at the input of the network and compare it to the cumulative spike count at the true decision neuron for four different models, as depicted in Figure 5. For illustrative purposes, we select four different English-speaking digit utterances: “1”, “6”, “7”, and “10”. The figures clearly show that the model without delay gradually increases its prediction as the input spikes come in and starts to do so as soon as input spikes start arriving. Conversely, for the other three models equipped with delay modules, the decision to increase spike count in the true neuron is delayed but then increases more quickly and reaches a higher level. This phenomenon arises from the different neurons introducing varying delays to the spikes, thereby providing the terminal neuron with multi-scale information. This may be interpreted as the VAD-enabled network aggregating all information in the spoken word before triggering a decision using all that information simultaneously. Moreover, we can observe that the models with delay typically have a total of 60 time step latency, which can be measured after the input is over. This is not only related to the delay itself but also to the choice of loss evaluation. As Shrestha et al. (2022) discussed, the spike-based negative log-likelihood loss results in early classification, even 1400 time steps faster than spike-rate based loss evaluation for NTIDIDIGITS datasets. However, the DL-128-SNN-Dloss generates the highest number of spikes for the true neuron compared to the other models, demonstrating its superior ability to learn characterizations.

**Figure 6:**
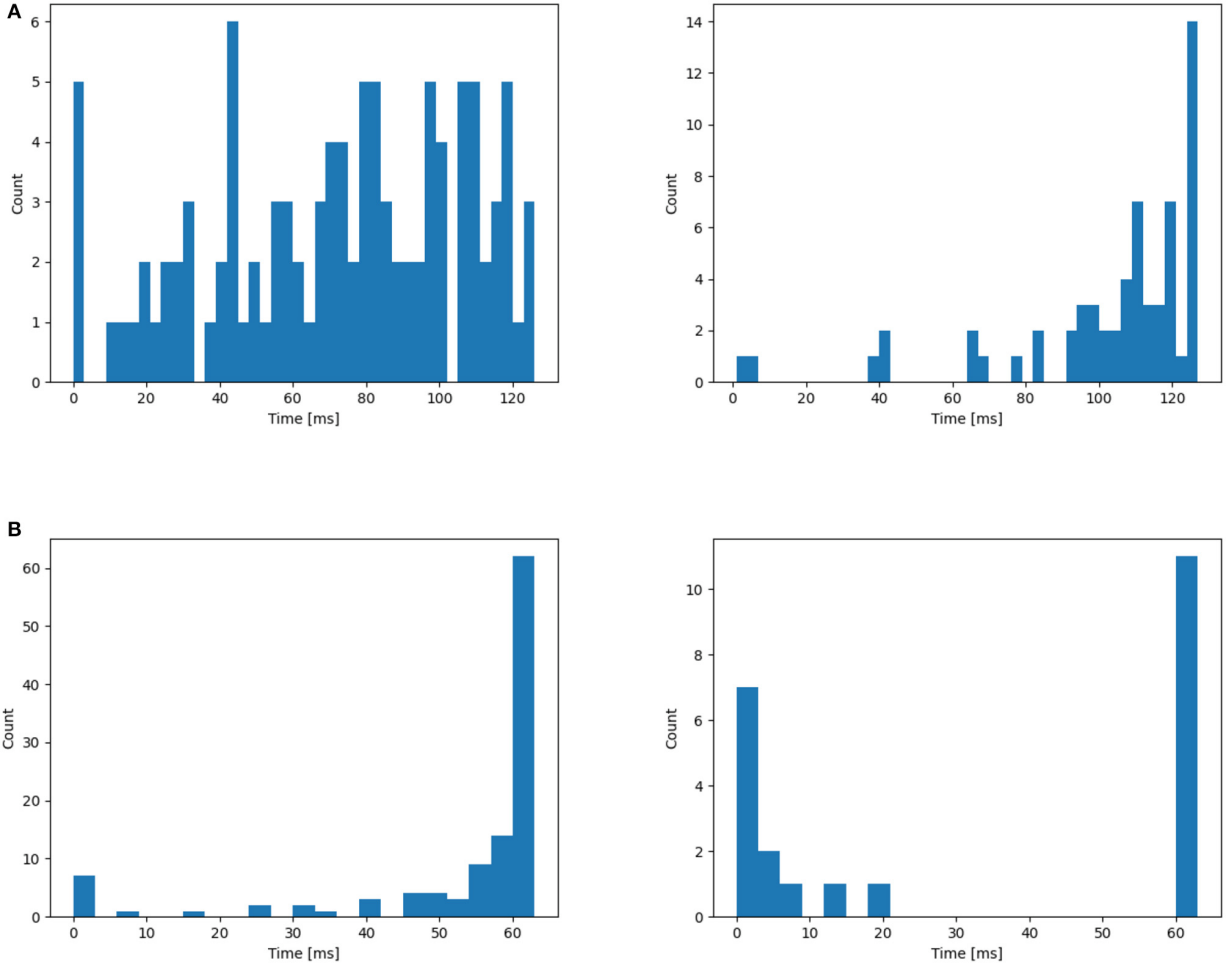
Application of the L2 regularizer on the distribution of time delay for **(A)** NTIDIDIGITS, **(B)** SHD. The initial distribution are all 0. From the left to right: First layer, second layer.

Subsequently, the L2 loss is employed to confine the range of delay values to provide a more uniform distribution. This leads to a reduction in delay values for some neurons (see Figure 6), aiming to reduce the total latency and investigate whether shorter delays contribute to a better classification system. This is achieved by applying the L2 regularizer to $\overset{N}{\underset{i=1}{\sum }}{\hat{d}}_{i}$. Nevertheless, as demonstrated in Table 4, the inclusion of the additional L2 loss results in a performance decline. This could indicate that the learned distributions achieved through these architectures may already be optimal within the current delay threshold, denoted as θ_*d*_.

### 3.6. Local skip-connection as compensation for loss of information in reset mechanism

**Figure 7:**
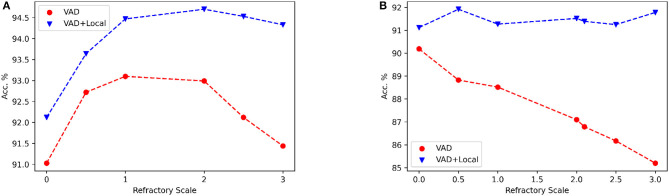
The influence of the different refractory scale α_*r*_ on accuracy is examined under “VAD” and “VAD+Local” architecture. “VAD” refers to the performance of using only the VAD module, while “VAD+Local” represents the performance using both VAD and local skip-connections. **(A)** NTIDIDIGITS dataset. **(B)** SHD dataset. For this experiment, we use two dense layers with 128 neurons.

The positive impact of local skip-connections on the reset mechanism becomes evident when modulating the refractory scale, symbolized as α_*r*_. We conduct a comparative analysis of performance between two distinct configurations: one labeled as VAD, which encompasses solely the delay model, and the other designated as VAD+Local, which additionally incorporates local skip-connections. As shown in Figure 7, the Local skip-connection maintains high performance across a wider range of refractory scales α_*r*_, while the performance with only the VAD module starts to decline with high values. This observation aligns with our earlier conjecture that larger values of α_*r*_ may induce information loss, as the neuron's potential struggles to recover efficiently. In contrast, the presence of local connections mitigates this loss by dynamically triggering spiking events among local neurons. Thus, our Local skip-connection diminishes sensitivity to parameter selection, potentially providing more flexibility to train SNNs for varied tasks, indicating that a consistent alpha value can be effective for different tasks.

## 4. Conclusion

In this study, we introduce several innovative components aimed at enhancing the performance of Spiking Neural Networks (SNNs): the learnable axonal delay module, combined with a local skip connection architecture, and augmented with an auxiliary suppressed loss. The variable axonal delay module plays a pivotal role in aligning spike timing, thereby enhancing the network's capacity for representation. The local skip-connection mechanism compensates for the information loss during the reset process. This enhances network dynamics and reduces the sensitivity to refractory scale tuning, making it more versatile. The inclusion of the suppressed loss works to suppress erroneous neuron firing, facilitating the SNN in making more accurate label distinctions. Importantly, these methods can be seamlessly integrated into the existing framework through the use of backpropagation algorithms.

We demonstrate that the proposed methods boost performance on two benchmark event-based speech datasets with the fewest parameters. Our methods highlight the immense potential of employing them in tandem with a cochlear front-end that encodes features of auditory inputs using spikes, creating a robust bio-inspired system. Our work emphasizes the importance of delving into different dynamic SNN architectures and learning algorithms for tasks involving datasets with rich temporal complexity.

In future work, it will be interesting to investigate the spike count distribution per layer and the total computational cost. Additionally, more exploration could be focused on latency by studying the influence of different loss evaluations and dynamic caps for axonal delays. Since current work mainly focuses on cochlear features with a bio-inspired approach, it would also be intriguing to apply these methods to visual tasks that involve inherent temporal information.

## Data availability statement

The original contributions presented in the study are included in the article/supplementary material, further inquiries can be directed to the corresponding author.

## Author contributions

PS: Conceptualization, Investigation, Software, Validation, Writing—original draft, Writing—review & editing. YC: Conceptualization, Investigation, Supervision, Writing—review & editing. PD: Supervision, Writing—review & editing, Conceptualization, Investigation. DB: Conceptualization, Funding acquisition, Investigation, Methodology, Project administration, Resources, Software, Supervision, Validation, Writing—review & editing.
